# Genetic loci contributing to hemophagocytic lymphohistiocytosis do not confer susceptibility to systemic-onset juvenile idiopathic arthritis

**DOI:** 10.1002/art.23270

**Published:** 2008-03

**Authors:** Rachelle Donn, Stuart Ellison, Rebecca Lamb, Thomas Day, Eileen Baildam, Athimalaipet V Ramanan

**Affiliations:** 1 University of ManchesterManchester, UK; 2 Royal Liverpool Children's HospitalLiverpool, UK; 3 Bristol Royal Hospital for ChildrenBristol, UK and Royal National Hospital for Rheumatic DiseasesBath, UK

## Abstract

**Objective:**

To investigate whether single-nucleotide polymorphisms (SNPs) within the genes *PRF1, GZMB, UNC13D*, and *Rab27a*, which are involved in natural killer cell dysfunction and known to contribute to the risk of hemophagocytic lymphohistiocytosis (HLH), confer an increased risk of susceptibility to systemic-onset juvenile idiopathic arthritis (JIA).

**Methods:**

Four SNPs across the *PRF1* gene locus, 5 for *GZMB*, 7 for *UNC13D*, and 11 for *Rab27a* were investigated using MassArray genotyping in 133 UK Caucasian patients with systemic-onset JIA and 384 ethnically matched unrelated control subjects. Additional control genotypes were accessed from the data generated by the Wellcome Trust Case Control Consortium.

**Results:**

No significant association was found between any SNP within the 4 selected loci and systemic-onset JIA, by either single-point or haplotype analysis.

**Conclusion:**

The results of this study demonstrate that genes involved in HLH do not confer a significant risk of association with systemic-onset JIA.

Systemic-onset juvenile idiopathic arthritis (JIA) constitutes 10–20% of all cases of JIA (1) but accounts for two-thirds of the mortality that occurs in these patients (2). The major cause of mortality over the last decade has been macrophage activation syndrome (MAS) or hemophagocytic lymphohistiocytosis (HLH). MAS is a severe, potentially life-threatening condition characterized by the excessive activation of well-differentiated macrophages. It results in fever, hepatosplenomegaly, lymphadenopathy, severe cytopenia, serious liver disease, coagulopathy, and neurologic involvement. MAS is usually seen with systemic-onset JIA and very rarely with the other subtypes of JIA (3,4).

A variety of triggers, including viral infections, active systemic disease, and drugs, have been implicated in the pathogenesis of MAS in patients with systemic-onset JIA (3). The true incidence of MAS in this subtype of JIA is not known. In one retrospective review of cases from a tertiary institution, MAS developed in 7 (6.8%) of the 103 children in whom systemic-onset JIA was diagnosed over a 20-year period (5). Those investigators, however, postulated that the true incidence of MAS might have been much higher, since mild cases of MAS are not always diagnosed. It is widely acknowledged by many pediatric rheumatologists that subclinical MAS is seen frequently (3,6,7). It has been speculated that MAS and systemic-onset JIA are simply “different ends of the same spectrum” (3,7). The presence of coagulation abnormalities and greatly elevated serum ferritin levels, both of which are characteristic features of MAS, in the majority of patients with active systemic-onset JIA is consistent with this idea.

It is increasingly recognized that MAS bears a close resemblance to a histiocytic disorder, secondary HLH, that is seen in a heterogeneous group of diseases, including infections, neoplasms, hematologic conditions, and autoimmune disorders (8). The current classification of histiocytic disorders categorizes hemophagocytic syndromes as either primary or secondary. Primary HLH includes a clearly familial form and a sporadic form without a clear family history that is nonetheless thought to be genetic in origin. Secondary HLH is seen in the context of a wide variety of conditions. Our 4 candidate genes were selected for investigation because, in up to 50% of patients with primary HLH, the underlying defect is caused by mutations in the genes encoding for perforin (*PRF1*; 10q22) or *UNC13* (*Caenorhabditis elegans* homolog of D [*UNC13D*]; 17q25.1) (9). Perforin is required to deliver granzyme B to target cells, after which an apoptotic program is initiated, leading to the demise of the target cells. Thus far, mutations within the gene encoding for granzyme B (*GZMB*; 14q11.2) have not been described in cases of HLH, but the functional role of *GZMB* suggests it is a valid candidate for genetic investigation. Finally, mutations in Ras-associated protein Rab 27A (*Rab27a*; 15q21) have been shown to play a causal role in the development of HLH in patients with Griscelli syndrome (9).

In a study by Grom et al, natural killer (NK) cell activity was shown to be low in patients with MAS (10). Some of these patients also had low levels of perforin expression, although genetic analysis did not identify the mutations that had been subjected to screening. Recently, NK cell dysfunction was investigated in a cohort of patients with pauciarticular JIA, polyarticular JIA, or systemic-onset JIA (11). The results of the study showed that in patients with systemic-onset JIA who had not yet had an episode of MAS, there was a decrease in NK cell function, similar to that seen in HLH. In the present study we therefore hypothesized that defective apoptosis in patients with systemic-onset JIA could be due to polymorphisms in the genes *PRF1, GZMB, UNC13D*, or *Rab27a*.

## PATIENTS AND METHODS

### Patients and controls

Blood samples were obtained from all subjects following the provision of informed consent. Ethics approval was obtained from the Multi-centre Research Ethics Committee (MREC 99/8/84) and the University of Manchester committee on the ethics of research on human beings (8/92/[i][b]). DNA samples were collected from patients with JIA as part of the British Society for Paediatric and Adolescent Rheumatology national repository for JIA. The diagnosis of JIA was classified according to the International League of Associations for Rheumatology classification criteria (12).

DNA samples from 133 UK Caucasian patients with systemic-onset JIA were available for genotyping. A population of unrelated and ethnically matched healthy individuals (n = 384) was used as the control group. All control subjects either were blood donors or had been recruited via general practitioner patient registries. In addition, genotyping data were obtained from an additional ∼3,000 healthy UK Caucasian subjects, who were studied as part of the Wellcome Trust Case Control Consortium (WTCCC; for details see the WTCCC Web site at http://www.wtccc.org.uk) (13); these data were used as an additional control. This resource was searched to identify overlap with the SNPs that had been genotyped or to find perfect proxys (r^2^ = 1) with the SNPs.

### Genotyping

Haplotype-tagging SNPs were selected from the HapMap database (on the HapMap Web site at www.hapmap.org), and additional informative SNPs were selected from the National Center for Biotechnology Information (NCBI) SNP database (on the NCBI Web site at www.ncbi.nlm.nih.gov/SNP). SNPs with a minor allele frequency (MAF) of ≥10% were selected; these were in *PRF1* (n = 3), *GZMB* (n = 5), *UNC13D* (n = 7), and *Rab27a* (n = 11). In addition, the A91V change in the *PRF1* gene was also studied (14). Genotyping was performed using MassArray high-throughput DNA analysis with matrix-assisted laser desorption ionization–time-of-flight mass spectrometry (Sequenom, San Diego, CA). Amplifications were conducted according to the manufacturer's protocol. Polymerase chain reaction primers and probe sequences are available from the corresponding author upon request.

### Statistical analysis

The raw data were “cleaned” prior to analysis, which allowed us to remove DNA samples that failed to genotype for ≥20% of the SNPs assayed, and also to remove the SNPs that failed detection in ≥20% of samples. Hardy-Weinberg equilibrium was determined using Stata version 8 software (College Station, TX) in the case and control groups separately. SNPs were removed from the analysis if deviation from Hardy-Weinberg equilibrium (*P* ≤ 0.05) was observed in the controls. Genotype counts in the control panel from the WTCCC (n = 2,938) were compared with those in our own control group. In the case of nonsignificant differences between the control groups, data were combined, and the total control population was compared with the patients with systemic-onset JIA.

For single-point analysis, associations were investigated by chi-square test or Fisher's exact test, using Stata version 8. *P* values less than or equal to 0.05 were considered significant. For multipoint analysis, linkage disequilibrium was calculated, and associations of varying-length haplotypes were tested using the expectation-maximization algorithm. This was implemented using HelixTree software (http://www.goldenhelix.com/index.jsp).

This study had 80% power to detect an odds ratio of ≥2.0, at the 5% significance level, for all of the SNPs studied except for the A91V change in *PRF1*, for which the study had 80% power to detect an odds ratio of ≥2.4, also at the 5% significance level. These levels were determined using Quanto software (http://hydra.usc.edu/GxE). Utilization of the WTCCC control genotype data enhanced the study power for certain SNPs (see Table 3), providing 82% power to detect an odds ratio of ≥1.8, again at the 5% significance level.

## RESULTS

Three SNPs, rs88581 (in *PRF1*), rs10873219 (in *GZMB*), and rs11630859 (in *Rab27a*), deviated from Hardy-Weinberg equilibrium in the controls, and were therefore excluded from any further analysis. Our genotyping data for *PRF1* captured 80% of the common SNPs (MAF ≥0.05), with an r^2^ value ≥ 0.8, that are present in the phase II CEPH HapMap resource (listed at www.hapmap.org). Similarly, the coverage was 88% of the common SNPs for *GZMB*, 86% for *UNC13D*, and 97% for *Rab27a*.

The SNP genotype frequencies were compared between the patients with systemic-onset JIA and our control panel of healthy individuals. No significant associations (*P* ≤ 0.05) were observed for any of the SNPs (Tables 1 and 2). Furthermore, no significant associations of haplotypes were seen for any of the loci studied

**Table 1:** *PRF1* and *GZMB* single-nucleotide polymorphism (SNP) genotype frequencies in patients with systemic-onset juvenile idiopathic arthritis (JIA) and controls

	Systemic-onset JIA			Controls			
			
SNP,genotype	Total sample size	Subjects with genotype, no.	Frequency of genotype, %	Total sample size	Subjects with genotyope, no.	Frequency of genotype, %	P*
*PRFI*							
rs3758562	118			356			
AA		55	46.6		166	46.6	
AG		54	45.8		162	45.5	
GG		9	7.6		28	7.9	0.99
A91V	107			329			
CC		104	97.2		311	94.5	
CT		2	1.9		17	5.2	
TT		1	0.9		1	0.3	0.25†
rs885822	120			354			
TT		40	33.3		130	36.7	
TC		65	54.2		171	48.3	
CC		15	12.5		53	15.0	0.55
*GZMB*							
rs7144366	109			346			
CC		38	34.9		132	38.1	
CT		58	53.2		159	46.0	
TT		13	11.9		55	15.9	0.36
rs8192917	118			359			
AA		72	61.0		217	60.5	
AG		40	33.9		130	36.2	
GG		6	5.1		12	3.3	0.65
rs2236338	110			348			
AA		69	62.7		213	61.2	
AG		35	31.8		119	34.2	
GG		6	5.5		16	4.6	0.86
rs2236337	119			357			
TT		72	60.5		217	60.8	
TC		40	33.6		127	35.6	
CC		7	5.9		13	3.6	0.56

* By chi-square test.

† By Fisher's exact test.

**Table 2:** *UNC13D* and *Rab27a* single-nucleotide polymorphism (SNP) genotype frequencies in patients with systemic-onset juvenile idiopathic arthritis (JIA) and controls

	Systemic-onset JIA			Controls			
			
SNP.genotype	Total sample size	Subjects with genotype, no.	Frequency of genotype, %	Total sample size	Subjects with genotype, no.	Frequency of genotype, %	P*
*UNC13D*							
rs3744010	116			352			
GG		63	54.3		215	61.1	
GA		49	42.2		123	34.9	
AA		4	3.5		14	4.0	0.39†
rs7223416	113			338			
CC		58	51.3		191	56.5	
CG		49	43.4		129	38.2	
GG		6	5.3		18	5.3	0.61
rs3744026	119			357			
TT		110	92.4		327	91.6	
TC		9	7.6		30	8.4	
CC		0	0		0	0	0.85†
rs17581728	117			354			
CC		65	55.6		224	63.2	
CT		48	41.0		117	33.1	
TT		4	3.4		13	3.7	0.30†
rs2290768	120			354			
CC		110	91.7		315	89.0	
CT		10	8.3		39	11.0	
TT		0	0		0	0	0.49†
rs2290769	116			357			
CC		67	57.8		223	62.5	
CG		46	39.7		121	33.9	
GG		3	2.5		13	3.6	0.52†
rs7210574	116			348			
TT		61	52.6		190	54.6	
TC		46	39.7		132	37.9	
CC		9	7.7		26	7.5	0.92
*Rab27a*							
rs11629613	102			317			
TT		78	76.5		246	77.6	
TC		23	22.6		68	21.5	
CC		1	0.9		3	0.9	0.95†
rs7167572	93			289			
CC		31	33.3		119	41.2	
CT		45	48.4		128	44.3	
TT		17	18.3		42	14.5	0.63
rs11071175	120			357			
AA		29	24.2		92	25.8	
AG		66	55.0		181	50.7	
GG		25	20.8		84	23.5	0.51
rs4261468	111			347			
AA		68	61.3		203	58.5	
AG		40	36.0		130	37.5	
GG		3	2.7		14	4.0	0.23†
rs24444043	107			332			
CC		30	28.0		100	30.1	
CT		52	48.6		172	51.8	
TT		25	23.4		60	18.1	0.44
rs2444039	117			353			
GG		41	35.0		139	39.4	
GT		61	52.1		178	50.4	
TT		15	12.9		36	10.2	0.35
rs7181237	115			353			
CC		73	63.5		196	55.5	
CG		36	31.3		131	37.1	
GG		6	5.2		26	7.4	0.26
rs9920165	114			349			
AA		92	80.7		276	79.1	
AG		26	17.5		71	20.3	
GG		2	1.8		2	0.6	0.11†
rs1050931	111			353			
CC		80	72.1		224	63.5	
CT		28	25.2		116	32.9	
TT		3	2.7		13	3.6	0.20†
rs1061824	119			359			
AA		81	68.1		227	63.2	
AG		35	29.4		120	33.4	
GG		3	2.5		12	3.4	0.20†

* By chi-square test.

† By Fisher's exact test.

For 7 of the SNPs included in this study, genotyping information from an additional 2,938 control subjects was available from the WTCCC (http://www.wtccc.org.uk). No individual within our own control panel had been studied as part of the WTCCC. None of the SNPs in this additional control group deviated from Hardy-Weinberg equilibrium. There were no statistically significant differences in the genotype frequencies between our control panel and this large set of control data for the 7 SNPs of interest. Therefore, the control groups were combined for comparison of their genotype frequencies with those found in the systemic-onset JIA group. Despite the increased number of controls, no SNP was found to be significantly associated with systemic-onset JIA (Table 3)

**Table 3:** *PRF1, GZMB, UNC13D,* and *Rab27a* single-nucleotide polymorphisms (SNPs) in patients with systemic-onset juvenile idiopathic arthritis (JIA) and combined controls*

		Systemis-onset JIA			Combined controls			
				
Gene, SNP	Perfect proxy SNP (r^2^ = 1)	1,1	1,2	2,2	1,1	1,2	2,2	*P*
*PRF1,* rs3758562	rs1099427	55 (46.6)	54 (45.8)	9 (7.6)	1,501 (45.9)	1,466 (44.8)	304 (9.3)	0.87
*GZMB,* rs8192917	rs10909625	72 (61.0)	40 (33.9)	6 (5.1)	1,968 (59.7)	1,141 (34.7)	185 (5.6)	0.95
*UNC13d,* rs7223416	rs8067076	58 (51.3)	49 (43.4)	6 (5.3)	1,442 (45.2)	1,438 (45.0)	313 (9.8)	0.19
*Rab27a*								
rs11071175	rs8032594	29 (24.2)	66 (55.0)	25 (20.8)	890 (27.1)	1,672 (50.9)	722 (22.0)	0.60
rs2444039	Original SNP in WTCCC	41 (35.0)	61 (52.2)	15 (12.8)	1,275 (38.7)	1,541 (46.9)	473 (14.4)	0.53
rs1050931	rs7165491	80 (72.1)	28 (25.2)	3 (2.7)	2,094 (63.7)	1,063 (32.3)	133 (4.0)	0.19
rs1061824	rs7165491	81 (68.1)	35 (29.4)	3 (2.5)	2,097 (63.6)	1,067 (32.4)	132 (4.0)	0.52

* Values are the no. (%) of subjects. 1 = wild-type allele; WTCCC = Wellcome Trust Case Control Consortium.

## DISCUSSION

Host survival depends on an immune system that can protect against infectious pathogens and provide control mechanisms that spare the host from immune damage. The condition known as MAS comprises a set of symptoms caused by the excessive activation and proliferation of T cells and well-differentiated macrophages. This activation is known to accompany the presentation of systemic-onset JIA. In many such cases, an overwhelming inflammatory reaction occurs, which can be fatal. Clinically, MAS resembles secondary HLH.

Genes contributing to the pathogenesis of HLH are beginning to be defined (9). We aimed to determine whether any SNP within these key genes conferred an increased risk of susceptibility to systemic-onset JIA. We did not attempt to study a population of patients with systemic-onset JIA having a known history of MAS. Such a patient population would be useful to examine genetically. However, collection of such a cohort would require collaboration with multiple centers worldwide, and there is the possibility that the numbers of patients involved would still be small and of limited informative value for genetic association studies. Furthermore, we do not know which, if any, of the patients with systemic-onset JIA included in the present study experienced MAS. Instead, we attempted to determine whether there is a shared genetic basis between MAS/HLH and systemic-onset JIA per se, recognizing that other genes and as yet unidentified environmental triggers are also required for the expression of the systemic-onset JIA phenotype.

No association was seen for any of the SNPs in each of the 4 genes considered. This remained the case despite our attempt to increase the study power by accessing additional control genotype data. We can therefore conclude that these SNPs do not contribute a substantial risk of susceptibility to systemic-onset JIA. Increasingly, it is becoming apparent that for complex conditions such as systemic-onset JIA, the proportional risk at any one locus is small, with odds ratios substantially lower than 1.8 becoming the norm (15). In this respect, our study remains underpowered. However, it does represent one of the largest systemic-onset JIA cohorts available for genetic investigation, and our study hypothesis was based on observations of both clinical and functional outcomes.

In conclusion, we could not detect an association between SNPs in *PRF1, GZMB, UN13D*, or *Rab27a* and an increased risk of susceptibility to systemic-onset JIA. Further studies, in larger cohorts of patients with systemic-onset JIA, are required to evaluate whether these genes contribute a more subtle risk of disease susceptibility than could be determined from this study.

## AUTHOR CONTRIBUTIONS

Dr. Donn had full access to all of the data in the study and takes responsibility for the integrity of the data and the accuracy of the data analysis.

**Study design.** Donn, Baildam, Ramanan.

**Acquisition of data.** Ellison, Day, Baildam, Ramanan.

**Analysis and interpretation of data.** Donn, Lamb, Day, Ramanan.

**Manuscript preparation.** Donn, Ramanan.

**Statistical analysis.** Donn, Lamb.
